# Integrating firearm storage and safety devices into health care

**DOI:** 10.1093/haschl/qxae105

**Published:** 2024-08-22

**Authors:** Christopher R Cogle, Anirudh B Venkatesh, Jaclyn M Hall

**Affiliations:** Department of Medicine, College of Medicine, University of Florida, Gainesville, FL 32610, USA; Department of Medicine, College of Medicine, University of Florida, Gainesville, FL 32610, USA; Department of Health Outcomes and Biomedical Informatics, College of Medicine, University of Florida, Gainesville, FL 32610, USA

**Keywords:** Medicaid, public health, violence, insurance

## Abstract

Millions of US children and adolescents live in homes with loaded firearms, with only half of these homes securing their guns. Firearm-related deaths among youth have doubled over the past decade, making firearms the leading cause of death for children and adolescents in the United States. The recent advisory by the US Surgeon General, identifying firearm violence as a public health crisis, underscores the urgent need for responsible firearm ownership, including safe firearm storage. However, the healthcare community currently lacks durable medical equipment (DME) codes for firearm storage devices, limiting the ability of healthcare providers to support responsible gun ownership. We propose the establishment of DME codes for firearm storage and safety devices, which would facilitate insurance coverage of these vital prevention measures. Durable medical equipment codes would empower physicians and other healthcare providers to integrate anticipatory guidance and lethal means counseling of firearm safety into routine care and support hospital- and community-based efforts to prevent firearm-related injuries and deaths among children and adolescents.

Today, millions of US children and adolescents play and grow in homes with loaded firearms.^1^ But in only half of these homes are the guns secured.^2,3^ The American Academy of Pediatrics (AAP) recommends that families do not keep guns in their homes.^4^ The presence of even a single household firearm is associated with an increased risk of youth suicide.^5^ Child firearms deaths have doubled over the past decade and are the leading cause of death of children and adolescents in the United States.^6^ In 2021, 4739 youth died by firearm, of which 1420 (30%) were suicide, 168 (3.5%) were accidental, and nearly all were from unauthorized access to a gun (CDC Wonder). Most youth firearm suicides occur in the home with a gun owned by a family member.^7^

On June 25, 2024, the US Surgeon General, Dr. Vivek Murthy, declared firearm violence a public health crisis.^8^ In his advisory, Dr. Murthy called upon gun control measures including safe and secure firearm storage. The Centers for Disease Control and Prevention has identified evidence-based policies that demonstrate a reduction in gun violence, including safe firearm storage in households with children and adolescents.^5,9^ However, firearm storage and safety devices are currently unrecognized by the healthcare community because, quite simply, there is no associated code. Thus, we urge for the establishment of durable medical equipment (DME) codes designed explicitly for coverage of firearm storage and safety devices.

## DME codes

DME codes are standardized identifiers used in health care to categorize and track medical devices and equipment that are necessary for patient care. These codes facilitate billing, reimbursement, and inventory management, ensuring that essential medical equipment is accessible to patients who need it. For example, DME codes for breast pumps encourage physicians to recommend and prescribe them, especially for mothers facing challenges with breastfeeding. Additionally, DME codes for mobility aids, like wheelchairs, encourage patients with mobility impairments to seek necessary devices. DME codes affect physician and patient behavior because they point to a piece of equipment, streamline the process for insurance approval, enable payment to equipment manufacturers and physicians, and reduce financial barriers for families in need.

Importantly, DME that is medically necessary, reusable, and acceptable for in-home use is eligible for coverage under most state Medicaid agencies, Medicare, and private health insurance plans.

State Medicaid agencies decide whether and how to cover new DME codes through a comprehensive evaluation process that considers medical necessity, cost-effectiveness, and potential impact on patient outcomes. The process typically begins when a manufacturer, patient advocacy organization, or a physician requests the state Medicaid agency to add the DME code to its fee schedule. Some state Medicaid agencies regularly review new codes after they are published by the Center for Medicare and Medicaid Services (CMS) in late November or early December. State legislatures may also direct their Medicaid agencies through statute or general appropriation acts to add services or supports for Medicaid recipients and state employees in a state health insurance plan. The Medicaid agency then reviews the clinical evidence, often published in peer-reviewed research reports, guidelines from organizations such as the CMS or the United States Preventive Services Task Force, and policy statements from professional medical associations such as the AAP. The agency also conducts a budget impact analysis, considering the price of the equipment, projected utilization, and expected savings from avoidance or reduction of costly health care such as emergency department visits, hospitalizations, and rehabilitation. Stakeholder input may be solicited from healthcare professionals and the public. This evaluation typically takes months to complete. Once the evaluation is complete, the agency may include the new code in its DME and Medical Supply Services fee schedule with limits on age, maximum fee, rental amount (if applicable), units, prior authorization requirement, and maximum number of units within a certain timeframe such as two units every year. Alternately, the state Medicaid agency may negotiate with its managed care health plans to add the DME code as a value-added benefit (also termed expanded benefit). A value-added benefit in Medicaid is an additional service or coverage offered by managed care organizations that goes beyond the standard benefits package to enhance patient care, at no extra cost to the state or beneficiary. Examples of value-added benefits in Medicaid are fall prevention equipment in the patient's home, wearable health technology, over-the-counter medication allowances, and non-emergency transportation to clinic appointments.

DME codes are also used by physicians and health systems to support various important administrative and clinical functions such as care coordination, patient safety, quality improvement programs, and compliance with laws and regulations.

## Firearm safety devices are medically necessary

Considering the ubiquity of firearms and the high number of firearm deaths to youth in the United States, storage and safety devices are medically necessary for children in homes with guns. These devices serve as vital equipment in preventing injury, disability, and death, making them medically necessary in buildings and facilities where children play and learn, such as homes of relatives, friends, and schools. Storing all firearms locked and unloaded and storing all ammunition locked and separate from firearms are each associated with reduced gun violence.^10^

In 2010, the Affordable Care Act established new rules on preventive health care, requiring both public and private payers to cover preventive services without cost-sharing by patients, and adopted the AAP’s *Bright Futures* guidelines promoting Safety and Injury Prevention.^11^ These guidelines promote evidence-supported preventive care and specifically recommend that pediatricians counsel patients and their parents about firearm safety and security. Thus, in the private examination room, when a physician identifies a heightened risk of injury from the potential for unauthorized firearm access, it is standard practice to have a shared decision-making and health education discussion about firearm storage and safety devices.^12^ Firearm storage and safety devices provide significant public health benefits, akin to seat belts and child safety seats in automobiles, which have significantly reduced the number of child deaths from motor vehicle accidents.

## Implementing DME codes for firearm storage and safety devices

By using DME codes for firearm safe storage, Medicaid, Medicare, and private payers will serve a significant role in responding to the US Surgeon General's advisory. First, physicians need to be supported in their patient screening for firearm risk, clinician-initiated anticipatory guidance discussions during well visits, and lethal means counseling when the patient's risk of suicide is high. Surveys indicate that most parents want their child's pediatrician to ask about firearms, yet only 13% of parents are asked about firearms in the outpatient setting and <1% are asked in the inpatient setting.^13,14^ It is a difficult discussion for physicians to initiate because of lack of time or concern for offending parents, of which 14% said they would be offended if advised by their physician on firearms.^14^ DME codes help physicians engage in this discussion, enabling them to approach the topic of firearm safety through a clinical context without seeming intrusive or out of scope. DME codes would give physicians a medically recognized safety intervention as a practical tool to guide conversations. For example, when a patient screens positive for risk of firearm injury, the physician could recommend specific safety devices covered by these codes, thereby providing actionable advice. In cases where the patient is at high risk of suicide, DME codes would support lethal means counseling by offering tangible safety solutions that could be easily documented and reimbursed.

Another implementation of DME codes could be inclusion in workplace wellness programs. By recognizing firearm safety as an integral component of employee well-being, these programs could offer incentives for employees to access and use DME-coded firearm storage devices, such as safes or trigger locks. This could be done through discounts, reimbursements, or health savings account contributions, making it financially easier for employees to adopt safe storage practices at home.

The DME codes could enable new population health management opportunities for health insurers, state Medicaid agencies, and state Departments of Health to rapidly respond to areas with higher rates of firearm-related incidents with education and DME-coded provision of safety devices. In cases when patients show signs of depression or other mental health conditions and screen positive for firearms in the home, clinicians can use DME codes for firearm safety equipment, in addition to other services integrating behavioral and mental health therapists, case managers, and community organizations, for the purposes of preventing suicide or homicide.

Currently, collaboration among health insurers, healthcare professionals, and community organizations on the issue of firearm safety is rare. However, DME-coded provision of devices will provide the necessary data for new collaborative efforts. Firearm safety devices may save states money by reducing the costly firearm-related injuries, including emergency care, hospitalizations, rehabilitation, and long-term treatments for physical injuries and mental illness arising from the trauma of gun violence. In state Medicaid agencies, the DME codes could start as a value-added benefit or expanded benefit before transitioning to a covered benefit. However, the approved DME codes are first needed in order to define, standardize, price, and hold accountable the benefit. Moreover, actuaries will need a coded service to properly value firearm storage and safety devices in their work to verify actuarially sound payment rates.

The US Surgeon General's advisory called for educating patients; however, healthcare professionals could also be educated on firearm storage and safety. The new DME codes could be accompanied by Continuing Medical Education or Continuing Education Units on firearm safety for healthcare professionals, which would include how to screen, counsel, and effectively use DME-coded firearm security devices to promote safety. Finally, physicians and health systems could choose firearm safety as a quality improvement initiative and new DME codes would enable monitoring effectiveness of these novel quality initiatives.

## Application for new DME code

In December 2023, we applied to the CMS to establish a new Healthcare Common Procedure Coding System Level II Code, for “Firearm Storage or Safety Device.” We proposed defining firearm storage and safety devices using a definition already stipulated in the Code of Federal Regulations, Title 18, US Code 921(a) (34), which states that the term *secure gun storage or safety device* means

“(A) a device that, when installed on a firearm, is designed to prevent the firearm from being operated without first deactivating the device; (B) a device incorporated into the design of the firearm that is designed to prevent the operation of the firearm by anyone not having access to the device; or (C) a safe, gun safe, gun case, lock box, or another device that is designed to be or can be used to store a firearm and that is designed to be unlocked only by means of a key, a combination, or other similar means.”

We envision two sub-categories of DME that align with this definition. The first subcategory includes devices that directly attach to the firearm, consistent with definitions (A) and (B), above. Example equipment includes firearm chamber safety flags, trigger and cable locks, or hammerlocks. The second subcategory includes devices that securely store firearms, consistent with definition (C), above. Example equipment includes firearm storage lockboxes, safes, lockers, or cabinets.

As of the date of this publication, our application is still under review at the CMS. Sometimes hearings are held in the process of new code promulgation. None have been scheduled yet.

## Conclusion

The establishment of DME codes for firearm storage and safety devices is a crucial step in addressing the US Surgeon General's advisory on firearm-related injuries and deaths. Creating these codes would facilitate the integration of firearm safety into routine healthcare practice, enabling physicians to have meaningful discussions with patients and their families about safe firearm storage. Moreover, these codes would allow for insurance coverage of safety devices, reducing financial barriers and promoting responsible gun ownership. The application for new DME codes is currently under review at CMS and represents a pivotal opportunity to standardize and support vital prevention measures.

## Supplementary Material

qxae105_Supplementary_Data

## References

[1] Gallup. Percentage of households in the United States owning one or more firearms from 1972-2023. In Statista. Accessed March 3, 2024. https://www.statista.com/statistics/249740/percentage-of-households-in-the-united-states-owning-a-firearm/

[2] Anestis MD , Moceri-Brooks J, Johnson RL, et al Assessment of firearm storage practices in the US, 2022. JAMA Netw Open. 2023;6(3):e231447. 10.1001/jamanetworkopen.2023.1447 36862408 PMC9982690

[3] Miller M , Azrael D. Firearm storage in US households with children: findings from the 2021 National Firearm Survey. JAMA Netw Open. 2022;5(2):e2148823. 10.1001/jamanetworkopen.2021.48823 35191973 PMC8864510

[4] Petty JK , Henry MC, Nance ML, Ford HR. Firearm injuries and children: position statement of the American Pediatric Surgical Association. *Pediatr.* 2019 Jul 1;144(1). 10.1016/j.jpedsurg.2019.03.001 31079862

[5] Davis A . US gun violence in 2021: An accounting of a public health crisis. Johns Hopkins Center for Gun Violence Solutions; 2023. Accessed March 3, 2024. https://publichealth.jhu.edu/2023/cdc-provisional-data-gun-suicides-reach-all-time-high-in-2022-gun-homicides-down-slightly-from-2021

[6] McGough M , Amin K, Panchal N, Cox C. Child and teen firearm mortality in the U.S. and peer countries. KFF. July 18, 2023. Accessed February 27, 2024. https://www.kff.org/mental-health/issue-brief/child-and-teen-firearm-mortality-in-the-u-s-and-peer-countries/

[7] Schnitzer PG , Dykstra HK, Trigylidas TE, Lichenstein R. Firearm suicide among youth in the United States, 2004–2015. J Behav Med. 2019;42(4):584–590. 10.1007/s10865-019-00037-0 31367924

[8] Murthy V . The U.S. surgeon general's advisory on firearm violence: a public health crisis in America. US Department of Health and Human Services; Published online June 25, 2024. https://www.hhs.gov/sites/default/files/firearm-violence-advisory.pdf 39042747

[9] Madigan S , Racine N, Vaillancourt T, et al. Changes in depression and anxiety among children and adolescents from before to during the COVID-19 pandemic: a systematic review and meta-analysis. JAMA Pediatr. 2023 Jun 1. 10.1001/jamapediatrics.2023.0846 PMC10152379 37126337

[10] Monuteaux MC , Azrael D, Miller M. Association of increased safe household firearm storage with firearm suicide and unintentional death among US youths. JAMA Pediatr. 2019;173(7):657–662. 10.1001/jamapediatrics.2019.1078 31081861 PMC6515586

[11] Hagan JF Jr , Shaw JS, Duncan PM, eds. Bright Futures: Guidelines for Health Supervision of Infants, Children, and Adolescents. 3rd ed. American Academy of Pediatrics; 2007. 10.1542/9781581102239

[12] Cannon AD , Reese K, Tetens P, Fingar KR. Preventable tragedies: findings from the #NotAnAccident index of unintentional shootings by children. Inj Epidemiol. 2023;10(Suppl 1):52. 10.1186/s40621-023-00464-3 37872595 PMC10594669

[13] Monroe KK , Fried SQ, Rubin A, et al Firearms screening in the pediatric inpatient setting. Hosp Pediatr. 2020;10(1):37–42. 10.1542/hpeds.2019-0040 31792099

[14] Garbutt JM , Bobenhouse N, Dodd S, Sterkel R, Strunk RC. What are parents willing to discuss with their pediatrician about firearm safety? A parental survey. J Pediatr. 2016;179:166–171. 10.1016/j.jpeds.2016.08.019 27639529 PMC5123916

